# Microbiomes of air dust collected during the ground-based closed bioregenerative life support experiment "Lunar Palace 365"

**DOI:** 10.1186/s40793-022-00399-0

**Published:** 2022-01-26

**Authors:** Jianlou Yang, Yuming Fu, Hong Liu

**Affiliations:** 1 Key Laboratory for Biomechanics and Mechanobiology of the Ministry of Education, Beijing Advanced Innovation Center for Biomedical Engineering, School of Biological Science and Medical Engineering, Beihang University, No. 37 Xueyuan Road, Beijing, 100191 China; 2 State Key Laboratory of Virtual Reality Technology and Systems, School of Computer Science and Engineering, Beihang University, Beijing, 100191 China; 3 International Joint Research Center of Aerospace Biotechnology and Medical Engineering, Beihang University, Beijing, 100191 China

**Keywords:** Bioregenerative life support system (BLSS), Lunar Palace 365, Microbiomes, Antibiotic resistance genes (ARGs)

## Abstract

**Background:**

Understanding the dynamics of airborne microbial communities and antibiotic resistance genes (ARGs) in space life support systems is important because potential pathogens and antibiotic resistance pose a health risk to crew that can lead to mission failure. There have been few reports on the distribution patterns of microbiomes and ARGs in space life support systems. In particular, there have been no detailed investigations of microbiomes and/or antibiotic resistance based on molecular methods in long-term confined bioregenerative life support systems (BLSSs). Therefore, in the present study, we collected air dust samples from two crew shifts, different areas, and different time points in the "Lunar Palace 365" experiment. We evaluated microbial diversity, species composition, functional potential, and antibiotic resistance by combining cultivation-independent analyses (amplicon, shot-gun sequencing, and qPCR).

**Results:**

We found that the bacterial community diversity in the Lunar Palace1 (LP1) system was higher than that in a controlled environment but lower than that in an open environment. Personnel exchange led to significant differences in bacterial community diversity, and source tracking analysis revealed that most bacteria in the air derived from the cabin crew and plants, but no differences in microbial function or antibiotic resistance were observed. Thus, human presence had the strongest effect on the succession of microbial diversity in the BLSSs.

**Conclusions:**

Our results highlight that microbial diversity in BLSSs is heavily influenced by changes in crew and is unique from other open and controlled environments. Our findings can be used to help develop safe, enclosed BLSS that meet the requirements of human survival and habitation in outer space. In addition, our results can further enhance our understanding of the indoor air microbial community and effectively maintain a safe working and living environment, including plant growth.

**Supplementary Information:**

The online version contains supplementary material available at 10.1186/s40793-022-00399-0.

## Background

Numerous space programs are rapidly advancing toward crewed deep space exploration, including constructing and utilizing a crewed lunar base and human exploration of Mars [1]. Among these programs, the United States will land at the Moon's south pole before 2024 and establish a sustainable environment there before 2028 [2]. Since 2015, the European Space Agency (ESA) has also vigorously advocated its concept of a "lunar village", which is a large-scale cooperative project that will also lead to a permanent settlement on the Moon [3]. Although lunar exploration itself can greatly benefit various scientific and technological fields [4], the Moon is expected to become a testbed for crewed missions to Mars. Building a safe and closed habitat will be necessary to exist off Earth for an extended time, one of the core components of which is a biological regeneration life support system (BLSS) [5, 6]. A BLSS is a small, balanced, and self-sufficient man-made ecosystem in which air, food, and water are recycled in a closed and isolated environment [7]. Chinese Lunar Palace 1 (LP1) is a ground-based BLSS testbed integrating efficient plant cultivation, animal protein production, urinary nitrogen recovery, and solid waste biotransformation [8]. LP1 can be used to prepare for various technical and scientific challenges in a closed and isolated extraterrestrial living space.

It is expected that microorganisms will inevitably coexist with extraterrestrial crew members. Indeed, the number of microorganisms exceeds that of human cells in our body, and each person releases millions of microorganisms per hour [9]. Any undertaking without microorganisms is impractical, immoral, and undesirable because microorganisms are crucial to our health [10]. For example, immune regulation-inducing microorganisms (known as "old friends") that evolved with mammals may have positive health effects [11], while pathogens (such as *Streptococcus pneumoniae*) have adverse effects on human health [12]. Therefore, proper management of the microbial community is crucial to the success of extraterrestrial missions.

The most obvious threat to crew health is pathogens. This risk is exacerbated by crew limitations and proximity, limited treatment options, increased microbial transmission under microgravity [13], limited sanitary conditions, increased potential virulence, reduced sensitivity of bacteria to antibiotics in space [14, 15], and reduced immunity of crew due to microgravity, radiation, and pressure [16, 17]. In addition, the establishment of resilient microbial communities in their habitats may become complex due to the lack of environmental microorganisms competing for the same niche with human-transmitted pathogens [18]. Although no life-threatening infections have been found during space flight so far, conditional pathogens, which are part of normal human-related microbial diversity, have been found on the international space station (ISS) [19]. These pathogens may have caused dozens of minor, off-Earth medical conditions, including urinary tract, upper respiratory tract, and subcutaneous skin infections [13, 17].

Importantly, bacteria can carry antibiotic resistance genes (ARGs). The World Health Organization [20] has stated that the abuse and misuse of antibiotics leads to antibiotic resistance of pathogenic bacteria, which is a new threat to modern public health [20]. ARGs can be transferred to pathogenic and nonpathogenic microorganisms in the environment through horizontal gene transfer, mainly involving the integration of ARGs into pathogenic bacteria through mobile genetic elements (MGEs), such as integrons and transposons [21]. ARGs and MGEs are widely distributed in different environments, including restricted and open environments [22]. For example, some ARGs [e.g., tet(K) gene resistant to tetracycline] are significantly positively correlated with specific antibacterial chemicals (e.g., triclosan) in dust collected from multifunctional sports and educational facilities [23]. In addition, Gandara et al. [24] have isolated antibiotic-resistant *Staphylococcus aureus* from indoor and outdoor air and found that these airborne bacteria are more concentrated indoors than outdoors [24]. Although the drug resistance of host-related flora and the emergence of preferentially drug-resistant pathogens in building environments are attracting increasing attention, the distribution of ARGs indoors, especially in a restricted environment, remains poorly understood. In addition, it is unknown how the distribution of resistance genes will change as the occupants of an isolated environment change.

Most of the information to date on the composition and dynamics of microbiota in a controlled environment has been acquired from ground simulation studies and space station-related studies, such as ISS [25], Mars 500 (520 days) [26], Concordia base in Antarctica (1 year) [27], inflatable lunar/Martian similar habitat (ILMAH) (30 days) [28], and the HI-SEAS IV mission (365 days) [9]. Previous studies have shown that, in these extreme environments, microorganisms mainly come from human skin. The microbial community of the ISS is highly similar to those existing in ground, confined indoor environments, though some studies have shown increased levels of ARGs and virulence gene factors, and the metagenomic sequences of human pathogens persist over time [29, 30]. A comparative analysis between ISS and a similar environment on Earth showed that the microbial composition on the environmental surfaces of the ISS is different [25]. It is important to note that these environments are ecosystems in which only people and microorganisms participate; there have been no detailed investigations on microbiomes and/or antibiotic resistance based on molecular methods in a BLSS, which is an isolated environment that integrates humans, plants, animals, and microorganisms.

In 2018, based on the advanced ground-based BLSS, Lunar Palace 1, developed by a team led by Beihang University, China, the Lunar Palace 365 experiment was completed successfully. The mission lasted 370 days. During this time, there were two groups of crew members, four in each group, including two men and two women. Over the course of the experiment, two-shift changes were carried out. The purpose of this study was to achieve long-term survival in a closed BLSS environment. However, the experiment also provided an opportunity to study the microbial changes resulting from different groups of crew members during their residence in LP1. In this setting, air represents a vehicle for the movement of microbes from one habitat to another. While there have been several studies of the microbial communities present on humans [31, 32] and plants [33, 34], few studies have attempted to characterize the airborne microflora in a BLSS. Here, we present the results of our study of the microbial air succession of Lunar Palace 365, which was carried out by monitoring the bacterial air flora in different locations when different groups resided in the habitat. In the closed indoor environment, air samples from designated locations were sampled at three stages during the shifts of the two groups of crew members. In addition to metagenomic sequencing of air sample flora, 16S rRNA amplicons were also sequenced from the air samples, and the absolute amounts of bacteria in air samples were determined by fluorescence quantitative PCR analysis. Our research provides new insights for maintaining the health of residents in a BLSS and furthers the development of future deep space habitation.

## Methods

### The Lunar Palace 1 habitat and Lunar Palace 365 project

In brief, LP1 is a ground-based BLSS, which functions as a biosphere to support crew members with basic living necessities. The biotechnology of the system regenerates oxygen, water, and food, allowing humans to survive in the confined space for long periods. The installation is located in Haidian, Beijing, China (116° 25′ 29″ E, 39° 54′ 20″ N) and occupies a total area of 160 m^2^ and a total volume of 500 m^3^. LP1 now has two plant cabins (PC; each 10 × 6 × 3.5 m^3^), one comprehensive cabin (CC; 14 × 3 × 2.5 m^3^) which contains 4 private bedrooms, a living room, a bathroom, an insect culturing room, and a solid waste treatment cabin (SC) [35].

**Fig. 1:**
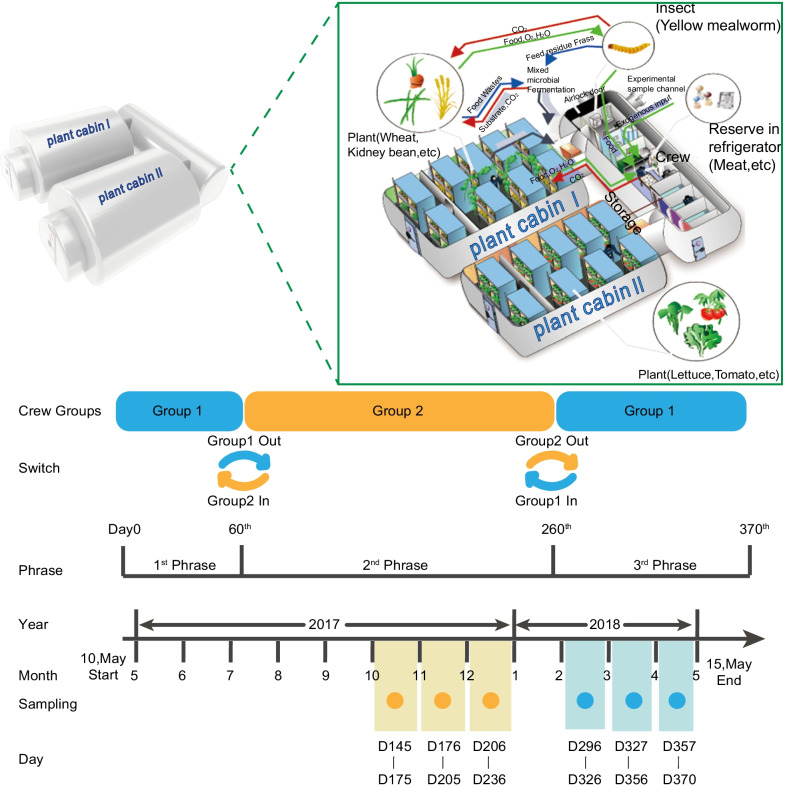
Timeline of the Lunar Palace 365 experiment. The schematic illustration indicates the key events during the confinement (above timeline), including the two shift change situations (two crew handover task simulations) and sampling dates of the 6 sampling events. Blue: Group 1; Yellow: Group 2. Light blue and light yellow areas indicate the sampling events during the different crew phases

This research was part of the Lunar Palace 365 project which was carried out in the Lunar Palace 1 (LP1). The Lunar Palace 365 project was launched on May 10, 2017, by the Institute of Environmental Biology and Life Support Technology, Beihang University. A total of eight volunteers were divided into two groups (G1 and G2; 2 females and 2 males each) that spent a total of 370 days in the LP1. The project was divided into three phases: the first phase lasted for 60 days with the four crew members of G1 (May 10 to July 10, 2017), the second phase lasted for 200 days with the four crew members of G2 (July 10, 2017, to January 26, 2018), and the third phase lasted for 110 days with the four crew members of G1 (January 26 to May 15, 2018) [36], the experimental design is shown in Fig. 1.

### Sampling procedures and DNA extraction

We collected 34 air dust samples. Sampling was executed by the same crew member to guarantee a consistent uptake of microbial particles. The air samples were collected by high-efficiency particulate absorbing (HEPA) filters (Xiaomi Air Purifier 2, 24 cm × 24 cm × 52 cm, Xiaomi Corporation, China) at three locations, including the plant cabins (I and II), comprehensive cabin, and solid waste treatment cabin. To enable the sample biomass to meet sequencing requirements, ambient air was sampled continuously over discrete 30-day periods onto a filter area of 450 cm^2^, maintaining a constant flow rate of 310 m^3^/h; sampling was performed in October, November, December of 2017, and March, April, and May of 2018, for a total of six sampling events. The purifier filter membrane was washed in phosphate-buffered saline (PBS; 1 × , CAS: SH30256.01, HyClone) under aseptic conditions, centrifuged, and the pellets were sent to Beijing QuantiHealth Technology Company Limited for DNA extraction using the DNeasy PowerSoil kit (QIAGEN) according to manufacturer’s instructions. The extracted DNA was examined by electrophoresis on a 1% agarose gel, and the DNA concentration and purity were determined with a NanoDrop 2000 UV–vis spectrophotometer (Thermo Scientific) and Qubit 3.0 fluorometer (Life technologies). The extracted DNA was divided into two parts, one for metagenomic analysis and the other for 16S amplicon analysis.

### Shot-gun metagenomics

After DNA extraction and preparation of the shot-gun library, all samples were sequenced using the Illumina sequencing HiSeq 2500 platform at Beijing QuantiHealth Technology Company for metagenome sequencing, yielding a total of 232.9G of raw sequencing data. The number of reads in the sample ranged from 30,394,642 to 145,747,924 with an average of 49,039,282, and all samples entered the bioinformatic data analysis process.

The biological information analysis process of metagenome sequencing entailed quality control of all original metagenome sequencing data through fastqc (v0.11.8) [37] software. First, all original sequencing reads were trimmed with trimmomatic software (v0.39-1, parameters: SLIDINGWINDOW:4:20 MINLEN:50) [38], and the reads with a quality value less than 20 and length less than 30 bp were filtered out. Clean reads were obtained by quality control. Finally, the filtered reads were compared with the host genome to remove the contaminated host sequence [39] using bowtie2 (v2.3.5.1, parameters: –very-sensitive – dovetail) to obtain high-quality, clean data. High-quality reads were de novo assembled with megahit (v1.2.9) [40] to obtain contigs, and evaluated with quast (v5.0.2) [41]. Based on the spliced contigs, prokka (v1.13.3) [42] gene annotation was used. The annotated genes were constructed with CD-HIT (v4.8.1, parameters: -aS 0.9 -c 0.95 -G 0 -M 0 -T 9 -g 1) [43], and salmon [44] was used for gene quantification. The obtained nonredundant reference gene set was compared using KEGG Orthology, Clusters of Orthologous Groups (COG), the ResFam resistance gene database, and the virulence factor database (VFDB) by DIAMOND (v0.8.36, parameters: blastp -v -sensitive -k 10) [45].

### 16S rRNA gene amplicons and sequencing

Another DNA aliquot was sent to Beijing Biomarker Technologies Corporation for 16S amplicon sequencing. Bacterial diversity was assessed by analyzing the V3 + V4 hypervariable region of the 16S rRNA gene amplicon sequences using two-step amplification. Amplification was performed with the following primer pair: forward primer, 338F, 5′-ACTCCTACGGGAGGCAGCA-3′, and the reverse primer, 806R, 5′-GGACTACHVGGGTWTCTAAT -3′ [46]. The 50 μl PCR reaction contained 1.5 μl of each primer, 1 μl dNTP, 10 μl Buffer, 0.2 μl Q5 HighFidelity DNA polymerase, 10 μl high GC content enhancer and 40 ng DNA template from the samples. The bacterial PCR reaction program was as follows: 95 °C denaturation for 3 min; 25 cycles of annealing at 50 °C for 45 s, extension at 68 °C for 90 s, extension at 68 °C for 7 min, and a final hold at 4 °C. The PCR products were collected and resolved on a 1.8% agarose gel, purified using a MinElute® PCR purification kit (Promega [Beijing] Biotech Co., Ltd. Beijing, China) according to the manufacturer's instructions, and quantified using QuantiFluorTM-ST (Promega [Beijing] Biotech Co., Ltd. Beijing, China). A library was constructed and sequenced, the final PCR product was purified, and the sequenced library was constructed after quantification and homogenization. Quality inspection was performed on the constructed library, and an Illumina HiSeq 2500 platform (Biomarker Technologies Co., Ltd., Beijing, China) was used to sequence the qualified library. Six biological replicates from each stage were sequenced. A total of 2,500,520 pairs of reads were obtained. After slicing and filtering the double-ended reads, 2,180,172 clean tags were generated. Each sample produced at least 37,711 clean tags, with an average of 66,066 clean tags.

The bioinformatics analysis process of amplicon sequencing was according to the tutorial of EasyAmplicon v1.09 [47]. The specific steps mainly include using the –fastq_mergepairs, –fastx_filter and –derep_fulllength subcommands in vsearch v2.15 [48] to double merge, quality control and de-duplication sequences, respectively. Then, the –cluster_OTU command of USEARCH v10.0 is used to cluster the nonredundant sequences into operational taxa (OTUs). Then, vsearch's –uchime_ref command was used to compare the feature sequences to the RDP database for further removal of chimeras. The –usearch_global command of vsearch generates a feature table. The characteristic sequences (OTUs/ASVs) were assigned taxonomically based on usearch's sintax algorithm and RDP [49] training set v16. In the environment of the R language v4.0.2, the vegan v2.5-6 [50] package was used for diversity analysis, and ggplot2 v3.3.2 package was used for data visualization [51]. PICRUSt2 (Phylogenetic Investigation of Communities by Reconstruction of Unobserved States; https://github.com/gavinmdouglas/q2-picrust2) and taxfun2 were used to predict potential phenotypes and functions.

### Bacterial quantification

The overall microbial load of air samples was determined by qPCR of the 16S rRNA gene. Bacteria-directed primers targeting the 16S rRNA gene, 1369F and modified 1492R [52] (Additional file 1: Table S1), were used for this analysis. Each 25 μl reaction consisted of 12.5 μl of 2 × iQ SYBR Green Supermix (Solarbio, Beijing), 1 μl each of forward and reverse oligonucleotide primers (10 μM each), and 1 μl of template DNA. qPCR runs were then carried out on a Bio-Rad CFX96 thermocycler with the following program: initial denaturation at 95 °C for 3 min, followed by 35 cycles of denaturation at 95 °C for 15 s, and combined annealing and extension at 55 °C for 35 s. Each sample was run in triplicate, the average and standard deviation were calculated based on these results. The number of gene copies in the samples was determined by running a standard curve, which was generated using serial dilutions (10^8^–10^2^) of *Bacillus pumilus* SAFR-032 16S rRNA gene [53]. The qPCR efficiency was ~ 98% for each run.

### Comparison of airborne bacteria in the LP system and in other environments

The sequence dataset and sample metadata are either shared by the original author, downloaded from public databases (such as NCBI sequence read archive [SRA] or the European nucleoside Archive), or obtained from the QIIME online database and FigShare repository. The QIIME online database is now used by the Qiita database (http://qiita.ucsd.edu). The International Space Station (ISS) airborne microbiological data set was obtained from the NCBI SRA repository (SRA #280254). The published Park ambient air microorganism project was obtained from the FigShare repository (https://doi.org/10.6084/m9.figshare.3362344). The microbial data set of classroom and outdoor ambient air is from the FigShare repository (http://dx.doi.org/10.6084/m9.figshare.157199). Plant indoor microbial data were obtained from the European nuclear Archive (PRJEB8807 [ERP009846]). Naturally ventilated indoor microorganisms were obtained from Qiita (Qiita study 1345). All studies were merged using BIOM table. QIIME and R were used for downstream data exploration and species diversity analysis.

Based on the sample species characteristic table and a previously described python script [54], SVM (support vector machine) [55] analysis was carried out to predict the restricted environment. Next, we randomly divided the data into two parts (training set and test set), conducted five cross-validations on the training set to adjust the SVM, and then analyzed the prediction error rate of the test set, using linear kernel prediction.

### SourceTracker analysis

To identify the relative contribution of different hypothetical sources to the bacterial community in the air of the LP system, two complementary methods were used, including fast expectation–maximization microbial source tracking (FEAST) [56] and indicator taxon analysis [57]. Both methods involved selecting five hypothetical source environments, including soil, plants, human skin, human mouth, and human/livestock feces, as they are considered potentially important sources of indoor microorganisms [12]. FEAST is an efficient method based on expectation maximization. This method takes the microbial community, source, and a group of separate potential source environments as inputs, and estimates the proportion of communities contributed by each source environment, which can then be applied to different sequencing data types. In short, soil-derived taxa were from Zhang et al. [58], plant-derived taxa were from Yi et al. [59], and data from human-related sources were from Costello et al. [60] and Zhu et al. [61]. At the same time, to determine the potential bacterial populations indicating these specific source environments, the indicator taxa of bacterial taxa were analyzed using the vegan2.3-5, indicspecies1.7.6, and labdsv1.8-0 packages in R3.1.3. Taxa with indicator values greater than 0.6 and *P* < 0.01 were selected as indicator taxa because they were considered to be highly correlated with a specific source environment [62].

### Identification of potential bacterial pathogens

The potential bacterial pathogens in the samples were determined according to the bacterial pathogen database reported previously [63], as per the classification table from the virulence factor database (http://www.mgc.ac.cn/VFs/), a bacterial pathogen database constructed from 557 pathogenic species (Additional file 1: Table S2) [64]. The 16S rRNA gene sequences of bacterial pathogens can be obtained from the NCBI (http://www.NCBI.NLM.NIH.gov/). The 16S rRNA gene sequence of each sample was compared with the 16S rRNA gene sequence of bacterial pathogens in the database, E-value < 1 × 10^−10^. The BLAST hit results were screened, and the potential bacterial pathogens with sequence similarity threshold > 99% were screened.

### Statistical analysis

For all statistical analyses, the significance level was set to *P* < 0.05 or the listed *P* value. R (4.0.2) was used for all statistical analysis and visualization (http://www.R-project.org/). We used PERMANOVA (permutational multivariate analysis of variance) [65] to test whether there were significant differences in microbial community structure in the sample group in the PCoA. EdgeR [66] (*P* < 0.05, FDR < 0.2) was also used to identify significant differences in the relative abundance of different groups among groups. Benjamini Hochberg corrected *P* values were based on Wilcoxon rank test or Kruskal Wallis test (significance threshold *P* < 0.05). Linear Discriminant Analysis (LDA) Effect Size (LEfSe) was used to determine which microorganisms were in differing abundance between groups [67]. LEfSe analysis was performed using a non-parametric factorial Kruskal–Wallis (KW) sum-rank test with an alpha value of ≤ 0.05, followed by an (unpaired) Wilcoxon rank-sum with an alpha score of ≤ 0.05, and a one-against-all strategy for multi-class analysis. To obtain the best discrimination output of taxa at the different locations in LP1, we used different algorithms (randomforest and XGBoost [68]), with the default parameters of the randomforest and XGBoost algorithm (package 'randomforest' and 'xgboost'). In R, the spearman rho value and the corresponding *P* value of the correlation analysis between microorganisms (genus level) and resistance genes were generated by the rcorr function. The adjust function was used to modify the *P* value using the Benjamin Hochberg method, and the Spearman correlation matrix constructed by Gephi [69] was used to evaluate the complexity of the interactions between microbiota and resistance genes.

## Results

### Bacterial population based on qPCR

**Fig. 2:**
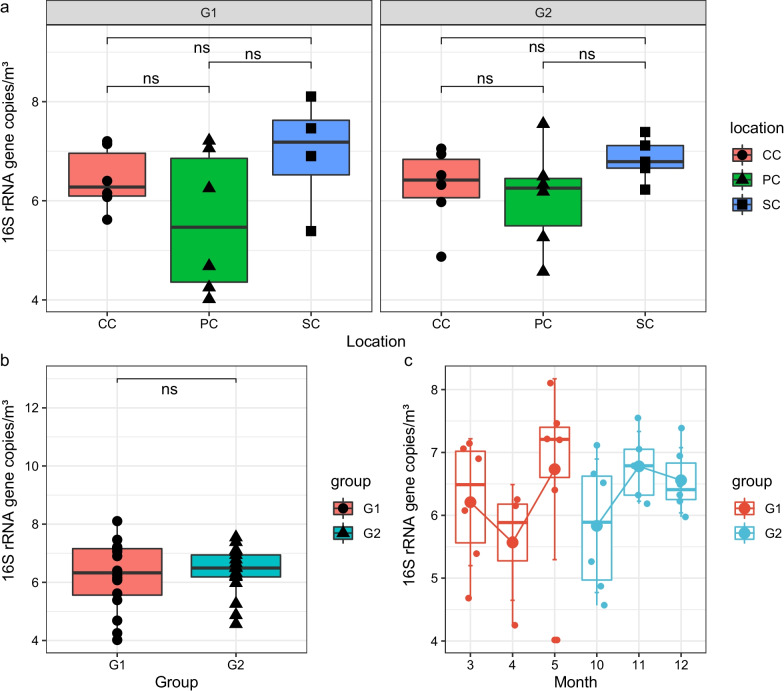
Bacterial population in the LP1 as estimated by qPCR. **a** Boxplot comparing the 16S rRNA gene (bacteria) copies in air dust samples from the indicated locations during the stays of the different crew groups of the Lunar Palace 365 experiment. **b** Boxplot comparing 16S rRNA gene copies across crew groups. **c** Boxplot comparing 16S rRNA gene copies over time. No statistically significant (*P* > 0.05) differences were observed, as per Student's t-test. CC: comprehensive cabin; PC: plant cabin; SC: solid waste treatment cabin; G1: crew group 1; G2: crew group 2. *P* value > 0.05 is indicated by "ns" for not significant

The bacterial load was measured by qPCR with the bacterial 16S rRNA gene as the target. The copy number of the gene showed a similar trend between the two groups: the bacterial load in the plant cabin air was relatively low, that in the comprehensive cabin was medium, and the solid waste treatment cabin was relatively high; however, there was no statistically significant difference between these values (Fig. 2a). In general, there was no significant difference in bacterial load between the different crew groups (*P* = 0.74; Fig. 2b). In G1, the average bacterial load of 1.43 × 10^7^ 16 s rRNA copies/m^3^ was higher than the average bacterial load of group G2 by 6.98 × 10^6^ copies/m^3^. Although the bacterial load fluctuated at different times (Fig. 2c), there was no increasing trend of bacterial load. In this study, the average number of bacteria in the air of the LP1 system was 1.05 × 10^7^ 16S rRNA copies/m^3^.

### Differences in the microbiome community between Lunar Palace I and other environments

**Fig. 3:**
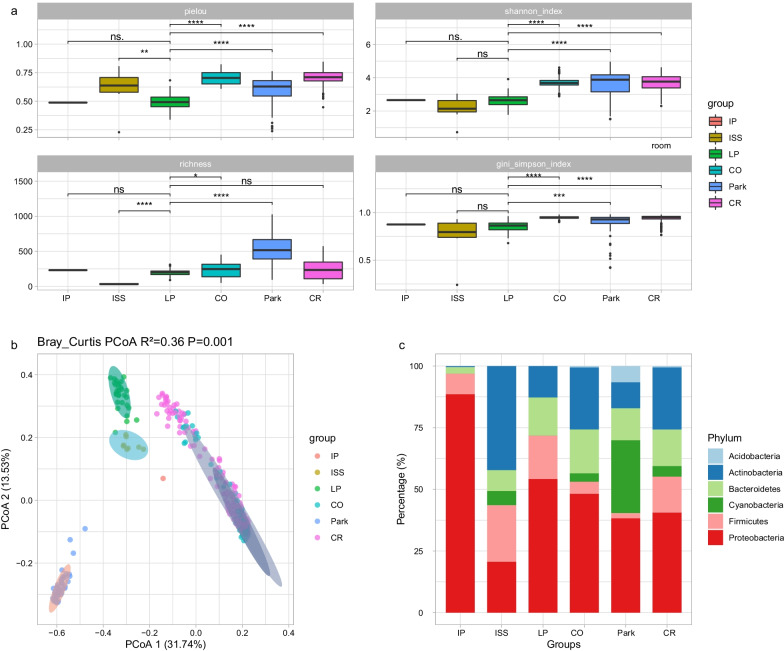
Composition of microorganisms and microbial diversity assessment in different environments. **a** Alpha diversity of bacteria communities. **b** Beta diversity estimates of the bacteria communities between different environments (PERMANOVA test [with 999 permutations], significance threshold, *P* < 0.05). **c** Relative abundance (%) of the major phyla present in the bacteria microbial communities. LP: Lunar Palace 1; ISS: International Space Station. IP: indoor with plants. CO: classroom outdoor. CR: university classroom. *P* value < 0.05 is represented as * and *P* value > 0.05 is indicated by “ns” for not significant

We compared the composition and diversity analyses of the ambient air in LP1 and the closed environment (ISS) and the open environments (classroom, park, classroom outdoor, and indoor with plants), based on 16S rRNA amplicon dataset. In terms of α diversity, whether examined using the Shannon index, Pielou evenness index, richness index, or Gini-Simpson index, there was no significant difference in the air microbial diversity between LP1 and the indoor with plants; however, the diversity was significantly different between LP1 and classroom, park and outdoor environments (Fig. 3a). In general, the diversity in LP1 was higher than that in the closed environment and lower than that in the open environments. As for β diversity, according to PCoA analysis based on Bray–Curtis distance, LP1 is quite different from the other environments. PC1 and PC2 account for 31.74% and 13.53% of the total variation, respectively. PERMANOVA test analysis revealed significant differences between the different environments (*P* = 0.001). In this type of analysis, the closer the distance, the more similar the species composition structure. Therefore, samples with high similarity of community structure tend to cluster together. The β diversity of air microorganisms in LP1 exhibits a uniqueness from both the closed environment and open environments (Fig. 3b). Using SVM analysis, we can predict from which unlabeled environment samples come with 84% accuracy, based only on microbiome composition (Additional file 2: Fig. S1a; F-1-score: ISS [0.50], LP [0.82], CO [0.79], Park [0.93), CR [0.85]), which further shows that air microorganisms in different environments have unique characteristics.

Through community composition analysis, Proteobacteria, Actinobacteria, Bacteroidetes, Cyanobacteria, and Firmicutes are the main bacterial phyla (Fig. 3c). In the indoor environment with plants, the relative abundance of Proteobacteria in air microorganisms was high. At the genus classification level, *Sphingomonas* was the main genus in the open environment, *Pseudomonas* was relatively abundant amongst the air microorganisms in the LP and ISS environments, while *Sphingomonas* was significantly lower than that in the other environments (Additional file 2: Fig. S1b).

### Effects of different crews of the Lunar Palace 365 experiment on the microbial community of LP1

**Fig. 4:**
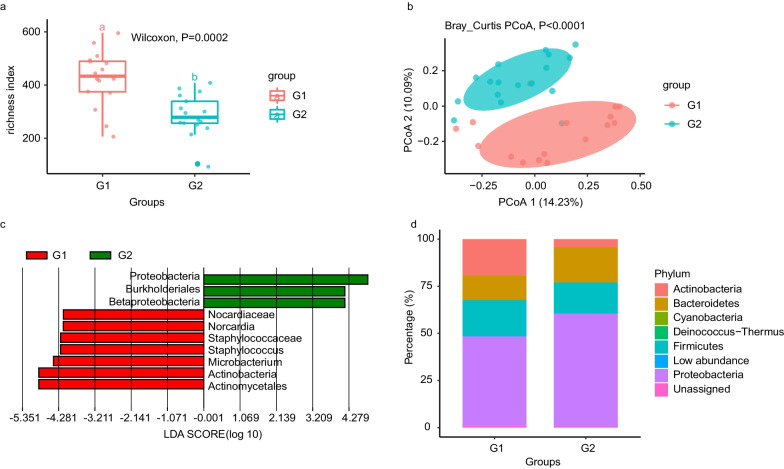
Microbial diversity estimates in the G1 and G2 groups. **a** Significant differences in richness diversity estimates of the microbial communities between the two crews. **b** Significant differences in PCoA based on Bray–Curtis estimates of microbial communities in the different habitation phases. **c** Lefse analysis identified differentially abundant genera between G1 and G2 (LDA significance threshold > 4.0). **d** Relative sequence abundance based on amplicon sequence of the bacterial phyla associated with G1 and G2

To better understand the effects of different crew groups on the microbial community, we compared the changes in microbial communities during the 200-day mission of group G2 and the 100-day mission of group G1. There was a significant difference in species richness between G1 and G2 for air samples, according to the 16S rRNA amplification and sequencing (richness index: *P* = 0.0021; Fig. 4a). We also analyzed the species from G1 and G2 by metagenome sequencing and found significant differences in α diversity (Shannon index: *P* = 0.0026; Additional file 2: Fig. S2). To further explore the impact of different crew compositions on the microbial community, we performed principal coordinate analysis (PCoA), based on Bray–Curtis distance, and observed a greater difference between the G1 and G2 samples (Fig. 4b).

We performed lefse analysis between the different crew groups (Kruskal Wallis; *P* < 0.05, LDA score > 4.0; Fig. 4c), which revealed significant differences in the relative abundance between G1 and G2. A histogram of the LDA value distribution shows that *Proteobacteria*, *Burkholderiales*, and *Betaproteobacteria* were enriched in the G2 group, while *Nocardiaceae*, *Nocardia*, *Staphylococcaceae*, *Staphylococcus*, *Microbacterium*, *Actinomycetes*, and *Actinobacteria* were enriched in the G1 group.

All samples were annotated with the RDP reference database. At the phylum classification level, four phyla (Proteobacteria, Firmicutes, Bacteroidetes, and Actinobacteria) were the main contributing bacteria (unclassified readings of bacterial phyla were removed from the sequencing data). The microbial composition in the G1 crew group was mainly composed of Proteobacteria (47.63%), Firmicutes (19.23%), Unassigned (0.81%), and Actinobacteria (19.12%), while the G2 group was mainly composed of Proteobacteria (59.99%), Bacteroidetes (18.69%), Unassigned (0.56%), and Firmicutes (16.48%; Additional file 1: Table S1, Fig. 4d). At the genus level, *Pseudomonas* (17.11%), *Acinetobacter* (9.74%), *Exiguobacterium* (3.22%), *Sphingobacterium* (3.88%), *Arthrobacter* (7.81%), *Microbacterium* (6.07%), and *Chryseobacterium* (2.00%) dominated the G1 crew group and *Pseudomonas* (19.85%), *Acinetobacter* (9.46%), and *Exiguobacterium* (7.61%) were prominent in the G2 crew group. (Additional file 1: Table S2, Additional file 2: Fig. S3).

A total of 284 species-level taxa were identified in the LP1 ambient air microorganisms by metagenomic sequencing. As expected, most communities were assigned bacteria (the average relative abundance of the whole data set was 99.7%), followed by viruses (0.23%) and archaea (0.045%). The core microbial composition was consistent with the results of the 16S rRNA sequencing at both the phylum and genus classification levels (Additional file 1: Table S3, Additional file 2: Fig. S4).

### Air microbial community by location in the Lunar Palace 365 experiment

Samples from different locations in the same crew group displayed the small differences (ANOSIM G1: r = 0.013, *P* = 0.38; G2: r = 0.092, *P* = 0.14 Additional file 2: Fig. S5d, e) compared with the samples from the same location during different crew groups (ANOSIM CC: r = 0.224, *P* = 0.029; PC: r = 0. 40, *P* = 0. 006; SC: r = -0.059, *P* = 0.58, Additional file 2: Fig. S5a, b, c). There was a significant difference in the air microbial community between two crew groups in the CC and PC, but there was no significant difference in the SC, likely because CC and PC are areas with dense occupant activities.

**Fig. 5:**
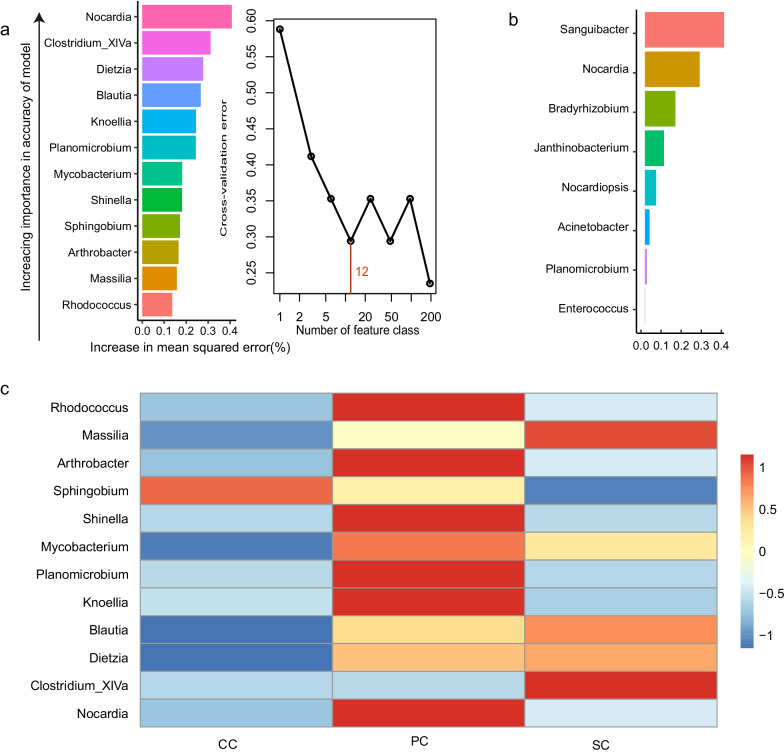
Bacterial biomarkers associated with the different locations in LP1. **a** A random forest approach was used to identify 12 genera, ranked in order of contribution from largest to smallest, associated with the indicated locations. **b** Identified biomarkers on genus levels based on XGBoost. **c** Heatmap showing the relative abundance of the 12 location-related biomarkers. CC, comprehensive cabin; PC, plant cabin; SC, solid waste treatment cabin

To identify important bacterial classifications as biomarker taxa related to different positions in LP1, we performed a tenfold cross-validation with 5 replicates to assess the importance of bacterial classification. When 47 important classes were used, the minimum cross-validation error was obtained. However, when 12 categories were used, the number of categories of the cross-validation error curve stabilized (Fig. 5a). Therefore, we defined these 12 categories as biomarker taxa in the model. Figure 5a lists the 12 most common bacterial taxa at the different locations in LP1 in order of importance. Similarly, based on the classification analysis using XGBoost, 8 bacterial taxa were determined as biomarkers, with a large contribution (Fig. 5b). Most biomarker taxa, such as *Nocardia* and *Rhodococcus*, exhibit a high relative abundance in the PC (Fig. 5c).

### Functional analysis based on 16S rRNA gene and metagenomic information

**Fig. 6:**
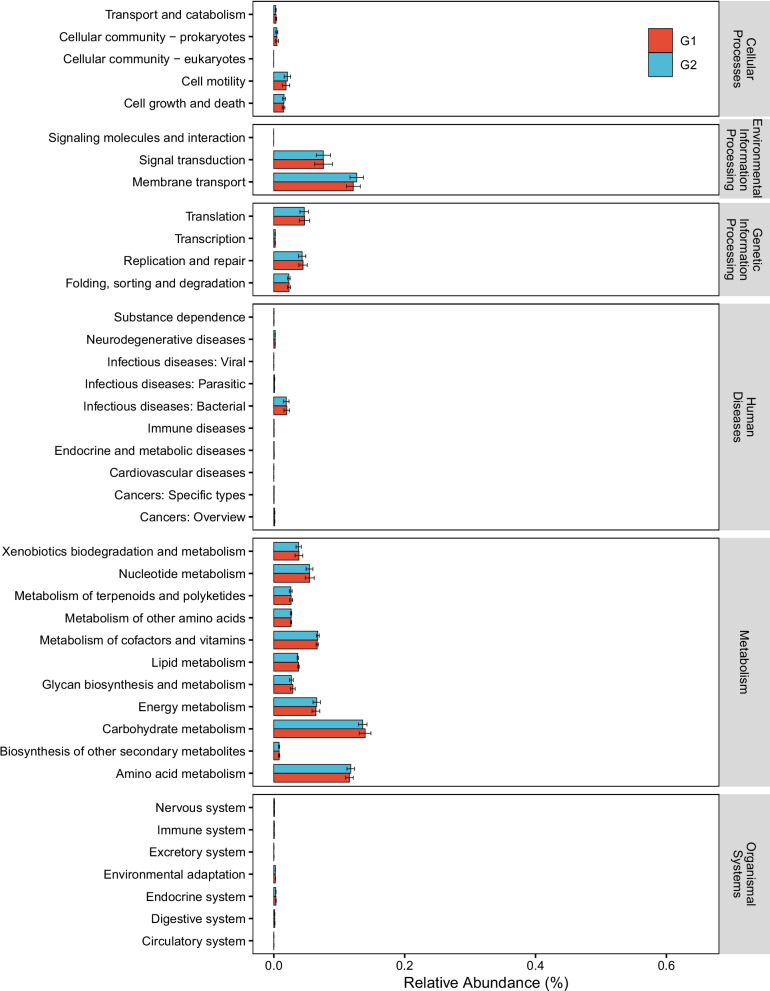
Functional difference analysis of KEGG pathway (level 2) between two groups using PICRUSt2

In the BLSS, the functional stability of each biological chain undoubtedly plays an important role in the stability of the ecosystem as a whole. Since the metagenome analysis can only be carried out on the mixed sample subset (due to the low biomass and sampling constraints), we initially used PICRUSt2 and tax4fun2 analysis based on 16S rRNA amplicon sequencing to predict the potential microbial metabolic capacity and functional redundancy. The PICRUSt2 results show that metabolism‐related pathways had the highest abundance at KEGG level 1. At KEGG level 2, the highest relative abundance was carbohydrate metabolism (Fig. 6). Between two crew groups, the bacteria was predicted to have no significantly differences capability of influencing genetic information processing, influencing environmental information processing, and human disease. Meanwhile, the functional diversity between G1 and G2 remained balanced without a significant difference based on level 3 of KEGG pathway analyses (Additional file 2: Fig. S6a, *P* = 0.081). The tax4fun2 functional redundancy analysis shows that nearly 6553 functions displayed a higher functional redundancy index in the G1 group, whereas only 350 functions had higher redundancies in the G2 group. The Relative functional redundancy indices of most functions were greatly reduced in the G2 group due to the decline of species diversity (Additional file 2: Fig. S6b, Fig. S2).

We performed metagenomic sequencing on the air samples to further verify whether there is a significant difference in functional diversity after a crew shift change. The results of level 3 of KEGG pathway analyses show no significant difference between the two groups as a whole (*P* = 0.4972; Additional file 2: Fig. S6c), consistent with 16 s rRNA amplicon prediction. The enrichment analysis results of KEGG show that the genes assigned to MCE-associated membrane protein, 4-phytase/acid phosphate, large subunit ribosomal protein L23AE, rhamnosyltransferase, and threonine synthase are abundant between the two groups, while biofilm formation—Pseudomonas aeruginosa, glycolysis/gluconeogenesis, starch and sucrose metabolism, Two-component system, Lipopolysaccharide biosynthesis was enriched and show differences between the two groups. This suggests that the microbial functions of the air dust samples are more concentrated in metabolism-related genes and functional pathways (Additional file 1: Tables S4 and S5), partially confirming the prediction result using PICRUSt2.

### Characteristics of antibiotic resistance genes in the Lunar Palace 365 experiment

**Fig. 7:**
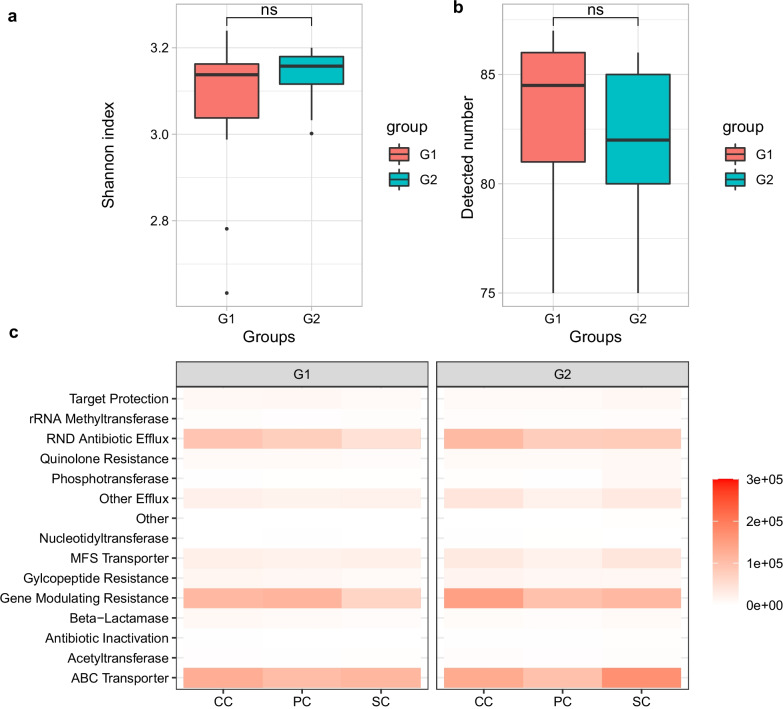
Detected number (**a**) and Shannon index (**b**) of ARGs found in the air dust samples. **c** Distribution of antibiotic resistance genes across samples as seen in metagenomics analysis. CC, comprehensive cabin; PC, plant cabin; SC, solid waste treatment cabin. *P* value > 0.05 is indicated by "ns" for not significant

Based on the differences between the two crews, and our interest in functions related to virulence and resistance, we investigated the antibiotic resistance genes of the microbiota (“resistome”) in air dust during the G1 and G2 groups' habitation in more detail. A total of 92 ARGs were observed in all 33 dust samples. The abundance and diversity of known resistance genes represent the resistome's non-characterized fraction in a given environment [70]. There was no significant difference in the number of ARGs detected (Fig. 7b) or the Shannon index among the different crews, with mean Shannon indices for G1 and G2 of 3.07 and 3.14, respectively (Fig. 7a). Among the different crew groups, the ARGs were mainly concentrated in four categories: (1) ABC transporter, (2) RND Antibiotic efflux, (3) Gene modulating resistance, and (4) MFS transporter (Fig. 7c).

To study the relationship between ARG expression and bacterial groups and determine whether bacterial genera help to explain the specific changes in ARG expression, we analyzed the Spearman correlation coefficient through the co-occurrence network (ρ) and *P* values (Additional file 2: Fig. S7) to visualize the correlation between ARG types and major genera. The correlation analysis revealed a strong correlation between ARGs and bacterial combinations (ρ > 0.7) and (*P* < 0.01). Among these relationships, *Arthrobacter* negatively correlated with *MexE*, and *Psychrobacter* negatively correlated with *TetY*, while the remaining were positively correlated.

### Potential bacterial pathogens in air samples from the Lunar Palace 365 experiment

Only six species of pathogenic bacteria were detected in all samples, including *Acinetobacter baumannii*, *Bacillus anthraci, Bacteroides thetaiotaomicron*, *Enterococcus faecalis*, *Escherichia fergusonii*, *Rickettsia conori*, based on the use of the 16S rRNA gene amplicon sequence BLAST against the bacterial pathogen 16S rRNA database [63]. Although 16S rRNA amplicon BLAST is an effective pathogen detection method, it still has the following limitations: Like all other PCR-based methods, inherent amplification bias in the PCR step cannot be excluded [71]. In addition, the relatively short sequence length (250 bp in this study) generally provides accurate taxonomic resolution at the genus level [72], therefore caution must be taken regarding the 6 pathogens mentioned above. Therefore, the nonredundant reference gene set was compared to the virulence factor database (VFDB) through metagenomic analysis. The results show that the most abundant virulence factors in all samples were flmH(3-oxoacyl-ACP reductase, ptxR(transcriptional regulator PtxR, scrC(sensory box/GGDEF family protein SrcC, sugC(ABC transporter-like protein), fbpC(ABC transporter, ATP-binding protein)( Additional file 1: Table S6). It must be noted that molecular assays, while a good approximation of the potential of virulence factors in an organism or microbial community, tend to overestimate this potential (since some virulence genes may not be expressed at all).

### Oral microorganisms of crew members are the main source of the airborne microbial community

**Fig. 8:**
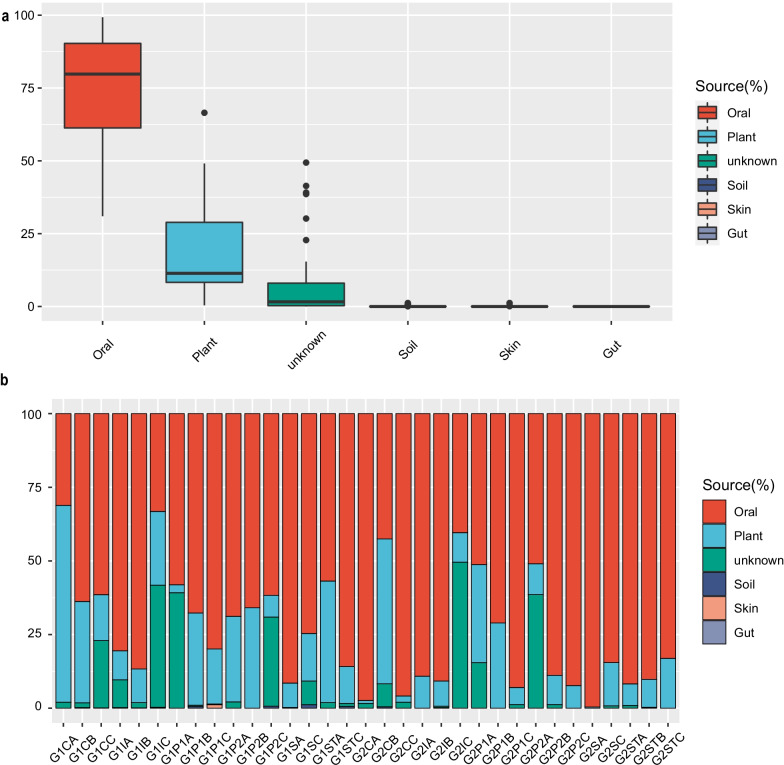
Sourcetracker analysis of airborne microbiota. **a** Abundance of bacterial genera identified by Dunn et al. as indicators of human and environmental sources. **b** FEAST predictions for microbiota sources

We used two methods to confirm that crew-related oral microorganisms are the main source of the microbial community in the air. First, we examined the relative abundance of indicator microorganisms identified by Dunn et al. [62] as being derived from human (skin, mouth, feces) and the environment (plant, soil). Among these organisms, the crew-related oral microorganisms were the main source of airborne microorganisms determined, while the plant rhizosphere microorganisms in the cabin were the second main source. The source of the most abundant organisms found on each air sample was the crew oral cavity (Fig. 8a). Next, we used FEAST analysis to determine whether crew oral microorganisms and plant rhizosphere microorganisms in the cabin are the most likely sources of the air microbial community. Most (> 80%) of the air microorganisms in the closed cabin came from human-related microbial groups, followed by plants (Fig. 8b).

## Discussion

At present, national and international space agencies are planning future missions to other celestial bodies such as the Moon or Mars. However, an autonomous life support system must be established for long-term residence off-planet. With extraterrestrial habitation for an extended period of time, microorganisms are expected to continue to accumulate. If they cannot be eliminated, the accumulation of microorganisms can lead to disease, posing a threat to crew health. The application of proven microbial disinfection techniques in a closed environment may greatly reduce the microbial population, but genetic material with the potential for biological pollution will also accumulate over time. Additionally, human well-being is inseparable from its microbiota. Thus, the dynamics of microbial communities inside and around human beings is an essential consideration for human long-term space flight and extraterrestrial settlement (such as a future Mars outpost). Earth-based models are essential for such research, including monitoring isolated inhabitants in a closed environment and the longitudinal microbial dynamics in the environment. Past work has examined many suitable model environments (not including the environment studied in the present work); however, there are essential differences in setting and research design. For example, the Concordia research station in Antarctica consists of independent buildings that can accommodate 16–32 people during sampling and has conducted 365 days of microbial monitoring [27]. The inflatable moon/Mars simulated habitat (ILMAH) in the United States provided 300 m^3^ space for three residents and conducted a 30-day survey [28]. Another example is the Mars500 habitat in Moscow, Russia, which includes four modules with a total volume of 550 m^3^ and can accommodate six participants for 520 days [26]. The most recent study was the HI-SEAS IV mission in Hawaii, a 1-year isolation study [9]. In our current study, air microorganisms in three critical sites in the LP1 system were characterized over two crew shifts, with sampling tasks lasting one year. This schedule enabled the examination of the temporal and spatial distribution of microbial populations.

The first question considered in our study is how the flora in the LP1 BLSS testbed compares with other closed, controlled, or open environments. DNA sequencing of 16S rRNA amplicons using primer set 338F and 806R revealed that the microbiota in the LP1 BLSS is significantly different from other closed, controlled, or open environments. This primer set was designed for the V3-V4 hypervariable region for DNA amplification, so that the amplified products could more easily cover this region and thus better reflect the sample diversity of the bacterial species [73]. 338F and 806R elicit very low amplification of non-target DNA, and have been widely used for 16S rRNA metagenomic primers [74, 75]. The use of the 338F and 806R primer pair also has been shown to reveal a high amplification efficiency of 96.58% for detection in complex intestinal flora [76]. Although PCR amplification bias introduced by primer selection is inevitable, the primer pair of 338F and 806R is well suited for BLSS microbiome analysis.

We found that the LP1 system had higher bacterial alpha diversity and different community structure than other closed habitats, but lower microbial diversity than open environments. One possible explanation is that the increased alpha diversity may be related to a richer nutrient environment for bacteria. We speculate that, compared with other controlled environments, LP1 has a greater degree of biological processes, such as plant growth and biological treatment of solid waste, providing richer nutrient sources for different bacteria. In addition, the average amount of total bacteria in the air was significantly lower than that recorded in other indoor environments, such as a flight cabin. The measured values ranged from 10^6^ to 10^7^ 16S rRNA gene copies/m^3^ [77]. At the same time, we found that the number of pathogenic bacteria in the air bacterial community in the LP1 system contains only six species. While the effects of indoor plants on indoor bacterial pathogens are unclear, since the crew members were the major source of bacteria in the habitat, the relatively low number of pathogenic species could be the result of the positive impact of indoor plants on human health [64]. Previous studies have shown the importance of occupants and home design (such as the introduction of indoor plants) in determining the structure of the indoor bacterial community. Introducing appropriate indoor plants is recommended as an effective method to improve air quality in different indoor environments [78]. These phenomena may explain the relatively small number of potential bacterial pathogens and total bacteria detected, either through the plants themselves and/or plant-related microbiota. This may also be the reason for the presence of a large number of unique bacterial OTUs in LP1. Consistent with previous reports [79], most bacteria in LP1 belong to Proteobacteria, Actinobacteria, Bacteroides, Cyanobacteria, and Firmicutes. Interestingly, compared with other closed environments, the relative abundance of *Proteobacteria* in the airborne microorganisms is higher in indoor environments with a large number of plants. At the genus level, *Sphingomonas* is the most abundant in the open environment. The relative abundance of *Pseudomonas* in air microorganisms in LP1 and the ISS environments is higher, and *Sphingomonas* is significantly lower than that in the open environment. Most species of *Proteobacteria* are common soil organisms and are usually endophytic and associated with plant rhizospheres [33]. The presence of these organisms is probably due to the large plant growth space in the LP1 system.

Several reports describe the microbial composition of simulated habitat environments used as substitutes for future human exploration using gene-targeted amplicon sequencing of the microbial population. One of these studies, ILMAH, detected a high abundance of *Staphylococcaceae*, *Corynebacteriaceae*, *Caulobacteraceae*, *Pleosporaceae*, and *Sporidiobolaceae* [28]. A similar closed system study, Mars500, found a high abundance of *Corynebacteriaceae*, *Burkholderiaceae*, and *Staphylococcaceae* [26]. The culturable microbial composition of a simulated underwater habitat was mainly composed of *Bacillus* (72%) and *Staphylococcus* (15%), indicating that the microbial composition in these simulated environments is also different from that in the LP1 environment.

Another initial objective of our study was to determine the effects of crew composition on bacterial communities and the dependence of the bacterial communities on sampling location. The flora of LP1 is strongly affected by having different residents. The diversity of the bacterial community decreased significantly from the G1 to the G2 group, indicating that the presence of passengers is an important determinant of the air microbial community in a BLSS. Previous studies have shown that in a closed physicochemical regenerative life support system (PCSS) habitat, the overall diversity of surface bacteria changes with the presence of humans [28]. Another study showed that the entry of personnel brought a large number of bacterial species into a closed BLSS living environment [80]. Our results confirm these findings and add new insights into how crew changes affect bacterial diversity. There are significant differences in the taxonomic composition of flora among healthy individuals, which is congruent with the different bacterial communities between the G1 and G2 groups.

Early studies did not find that the change in the indoor bacterial community positively correlates with human presence [81]. These conflicting observations may be related to whether the space is an open or closed system. Previous studies have shown that bacterial communities in open and common indoor spaces are mainly affected by outdoor air bacteria [82]. In addition, in contrast to the ISS [83], the LP1 ambient air flora comes from crew oral and plant rhizosphere-related microorganisms rather than human skin. Various reports have shown that in a strictly controlled environment, the presence of humans is the most common source of microorganisms, and human transmission of microorganisms depends on their activities and time in closed habitats. Generally, 10^6^ to 10^7^ microorganisms are turned over on the skin every day; sweating, coughing, or speaking will excrete 10^3^ to 10^4^ microorganisms, which once again is consistent with the assertion that human presence is the dominant contributor to the microbial community on indoor surfaces [18]. Most notably, the impact of crew composition on the bacterial community was dependent on sample location. There were significant differences in air bacterial communities in the CC and PC between G1 and G2, but there was no significant difference in SC.

Moreover, we found that the total number of bacteria in the PC was less than that in the CC and SC. Thus, the effect of the large plant growth area on the PC air bacterial community outweighed the contribution of the occupant change. The analysis of bacterial biomarkers at different positions in LP1 further confirmed this conclusion. *Nocardia* and *Rhodococcus*, as biomarkers distinguished the different sites, showed high relative abundance in the PC. *Nocardia* belongs to *Actinomycetes* and is abundant in plant endophytes. This genus has the potential to be a plant growth promoter and biological control agent [84], while most *Rhodococcus* species play important roles in promoting plant growth [85, 86].

Surprisingly, the microbial community in the samples contained a certain number of known ARGs, with diversity and abundance that are much lower than in other indoor environments [64]. These results imply that air in a confined environment is a neglected route of transmission and a reservoir of antibiotic resistance. This view is partially supported by some air-based studies, which propose that air transmission is an understudied pathway for the transmission of antibiotic resistance in various environments [87]. In our study, the reason behind the high diversity and richness of ARGs in the air may be the accumulation of antibiotic-resistant bacteria from the indoor environment. However, we do not have any information related to antibiotics, such as the antibiotic treatment history of the crew before sampling and the degree of medical environment exposure of the crew members prior to the experiment. Future studies should take these factors into account to better explain why confined room environments contain ARGs. The ARGs were mainly concentrated in four categories: (1) ABC transporter, (2) RND antibiotic efflux, (3) gene modulating resistance, and (4) MFS transporter, indicating the possibility of their transmission through the air and/or horizontal transfer. In contrast to the bacterial community, crew composition is unlikely to drive the structure of ARGs in the environment because no significant differences were detected in ARGs between the crew groups. Actinomycetes*,* Firmicutes, and Proteus contributed the most to the structural variation of related ARGs in the air. Actinomycetes are famous for producing antibiotics and are considered a common source of ARGs because they usually carry a variety of ARGs and have a variety of drug resistance mechanisms [88]. In addition, we observed that there was a negative correlation between *Arthrobacter* and *MexE*, and *Psychrobacter* and *TetY*, with the rest of the correlations positive. Therefore, we can infer that the transfer of these bacterial phyla possibly plays a major role in forming antibiotic-resistant structures in the air microbiome. The network analysis results further support this finding, which can be partially explained by the hypothesis that microorganisms shedding from the crew are the source of ARGs. Another possible explanation is that antibiotic selection pressure caused by human activity, such as antibiotic treatment of crew members, may directly affect the reservoir of resistance genes.

The current study has some limitations and provides meaningful information, albeit incomplete, for future attempts to maintain a safe microbial environment for human outposts on the Moon or Mars. The information on all the microbial sources is limited, and only a limited number of crew members were selected to monitor the transfer of potential microorganisms and ARGs into the environment. Additional human volunteers will help to conclude the contribution of personal health. Finally, the entire infrastructure and environment is a partial simulation of a bona fide extraterrestrial crewed mission.

## Conclusion

In conclusion, this study expounds the air microbiota and drug resistance in a long-term closed BLSS. The results highlight the specificity and distribution characteristics of the air microbial community and resistance genes in a BLSS across a change of crew members. Although we have begun to characterize the microbiology, further work is needed to determine the viability of the observed microbial community, because the detected functional and resistance genes are not necessarily indicative of phenotypes in this environment. This information can provide further insights into the occupant health risks associated with the spread of potential pathogens and antibiotic resistance in confined environments. Future work should also expand existing methods and findings, including microbiome and antibiotic resistance data from other closed and open environments, to comprehensively understand the differences between microbiota and antibiotic resistance caused by human factors. In addition, our results also expand our knowledge of indoor air microbial communities, which is essential to maintain safe working and living environments effectively.

## Supplementary Information

**Additional file 1: Table S1.** Main taxonomy at the phylum level. **Table S2.** Main taxonomy at the genus level. **Table S3.** Relative abundance of bacterial flora based on metagenomic sequencing. **Table S4.** Difference analysis results of KEGG Orthology (top 20). Table S5: Enrichment analysis results of KEGG Pathway Analysis (Top 10). Table S6. VFDB based on metagenome sequence

**Additional file 2: Figure S1.** (a) Comparison of microbial composition in different environments, according to SVM analysis. (b) Relative abundance (%) of the major genera present in the fungal microbial communities. LP: Lunar Palace 1; ISS: International Space Station. IP: indoor with plants. CO: classroom outdoor. CR: university classroom. **Figure S2.** Significant differences in richness diversity estimates of the microbial communities between the two crews. **Figure S3.** Relative sequence abundance, based on amplicon sequencing, of bacterial genera associated with G1 and G2. **Figure S4.** Relative sequence abundance, based on metagenome sequencing, of bacterial phyla (a) and genera (b) associated with G1 and G2. **Figure S5.** Comparison of community diversity among the different occupant groups and different sampling locations. (a) Comparison of community diversity among the different occupant groups in the CC. (b) Comparison of community diversity among the different occupant groups in the PC. (c) Comparison of community diversity among the different occupant groups in the SC. (d) Comparison of community diversity among the different sampling sites during the G1 group. (e) Comparison of community diversity among the different sampling sites during the G2 group. CC, comprehensive cabin; PC, plant cabin; SC, solid waste treatment cabin. **Figure S6.** Microbial functional diversity estimates for the two shifts. (a) Functional diversity between the two crew groups (no significant difference). (b) Functional redundancy index. (c) Functional diversity analysis based on metagenomics between the two crew groups. **Figure S7.** Correlation network analysis between ARGs and the airborne microbiome (*P* < 0.05, Spearman’s coefficient > 0.4). Grey lines between the nodes indicate positive connections between the genera, while red lines indicate negative correlations.

## Data Availability

The dataset of the 16 s rRNA sequencing and metagenome sequencing data reported in this paper has been deposited in the Genome Sequence Archive (Genomics, Proteomics & Bioinformatics 2017) in the National Genomics Data Center (Nucleic Acids Res 2020), the Beijing Institute of Genomics (China National Center for Bioinformation), Chinese Academy of Sciences, under accession number CRA004389 (metagenome) and CRA004384 (amplicon), which are publicly accessible at https://bigd.big.ac.cn/gsa. All other data are available from the GitHub site (https://github.com/YJL900223/LP_air).

## Abbreviations

ARGs: Antibiotic resistance genes
BLSSs: Bioregenerative life support systems
LP1: Lunar Palace1
ESA: European Space Agency
ISS: International space station
MGEs: Mobile genetic elements
ILMAH: Inflatable lunar/Martian similar habitat
PC: Plant cabins
CC: Comprehensive cabin
SC: Solid waste treatment cabin
G1: Group1
G2: Group2
HEPA: High-efficiency particulate absorbing
COG: Clusters of Orthologous Groups
VFDB: Virulence factor database
SVM: Support vector machine
FEAST: Fast expectation–maximization microbial source tracking
PERMANOVA: Permutational multivariate analysis of variance
PCoA: Principal coordinate analysis
IP: Indoor with plants
CO: Classroom outdoor
CR: University classroom
