# Supplementary material for: Microbiomes of air dust collected during the ground-based closed bioregenerative life support experiment "Lunar Palace 365"
Source: Environ Microbiome. 2022 Jan 26;17:4. doi: 10.1186/s40793-022-00399-0 (PMC8793263; doi:10.1186/s40793-022-00399-0)
Supplement: Supplementary file 2 — Additional file 2: Figure S1. (a) Comparison of microbial composition in different environments, according to SVM analysis. (b) Relative abundance (%) of the major genera present in the fungal microbial communities. LP: Lunar Palace 1; ISS: International Space Station. IP: indoor with plants. CO: classroom outdoor. CR: university classroom. Figure S2. Significant differences in richness diversity estimates of the microbial communities between the two crews. Figure S3. Relative sequence abundance, based on amplicon sequencing, of bacterial genera associated with G1 and G2. Figure S4. Relative sequence abundance, based on metagenome sequencing, of bacterial phyla (a) and genera (b) associated with G1 and G2. Figure S5. Comparison of community diversity among the different occupant groups and different sampling locations. (a) Comparison of community diversity among the different occupant groups in the CC. (b) Comparison of community diversity among the different occupant groups in the PC. (c) Comparison of community diversity among the different occupant groups in the SC. (d) Comparison of community diversity among the different sampling sites during the G1 group. (e) Comparison of community diversity among the different sampling sites during the G2 group. CC, comprehensive cabin; PC, plant cabin; SC, solid waste treatment cabin. Figure S6. Microbial functional diversity estimates for the two shifts. (a) Functional diversity between the two crew groups (no significant difference). (b) Functional redundancy index. (c) Functional diversity analysis based on metagenomics between the two crew groups. Figure S7. Correlation network analysis between ARGs and the airborne microbiome (P < 0.05, Spearman’s coefficient > 0.4). Grey lines between the nodes indicate positive connections between the genera, while red lines indicate negative correlations. [file 40793_2022_399_MOESM2_ESM.docx]

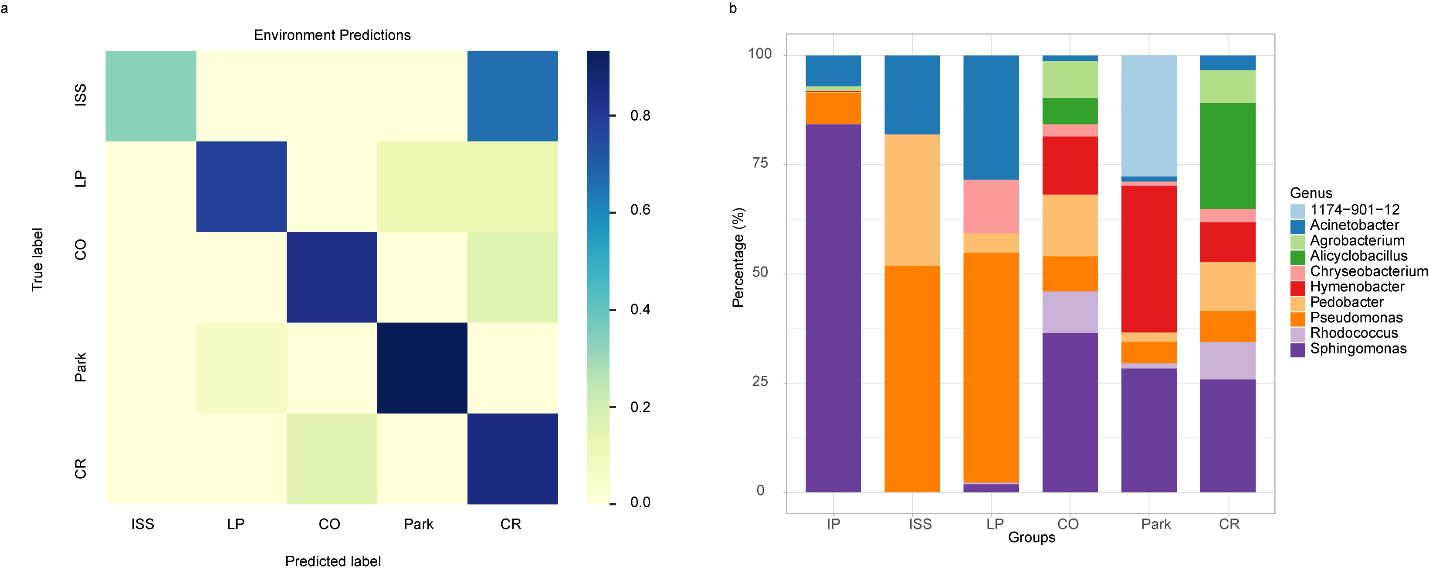


**Figure S1.** **(a)** **Comparison of microbial composition in different environments, according to SVM analysis.** **(b)** **Relative abundance (%) of the major genera present in the fungal microbial communities.** LP: Lunar Palace 1; ISS: International Space Station. IP: indoor with plants. CO: classroom outdoor. CR: university classroom.


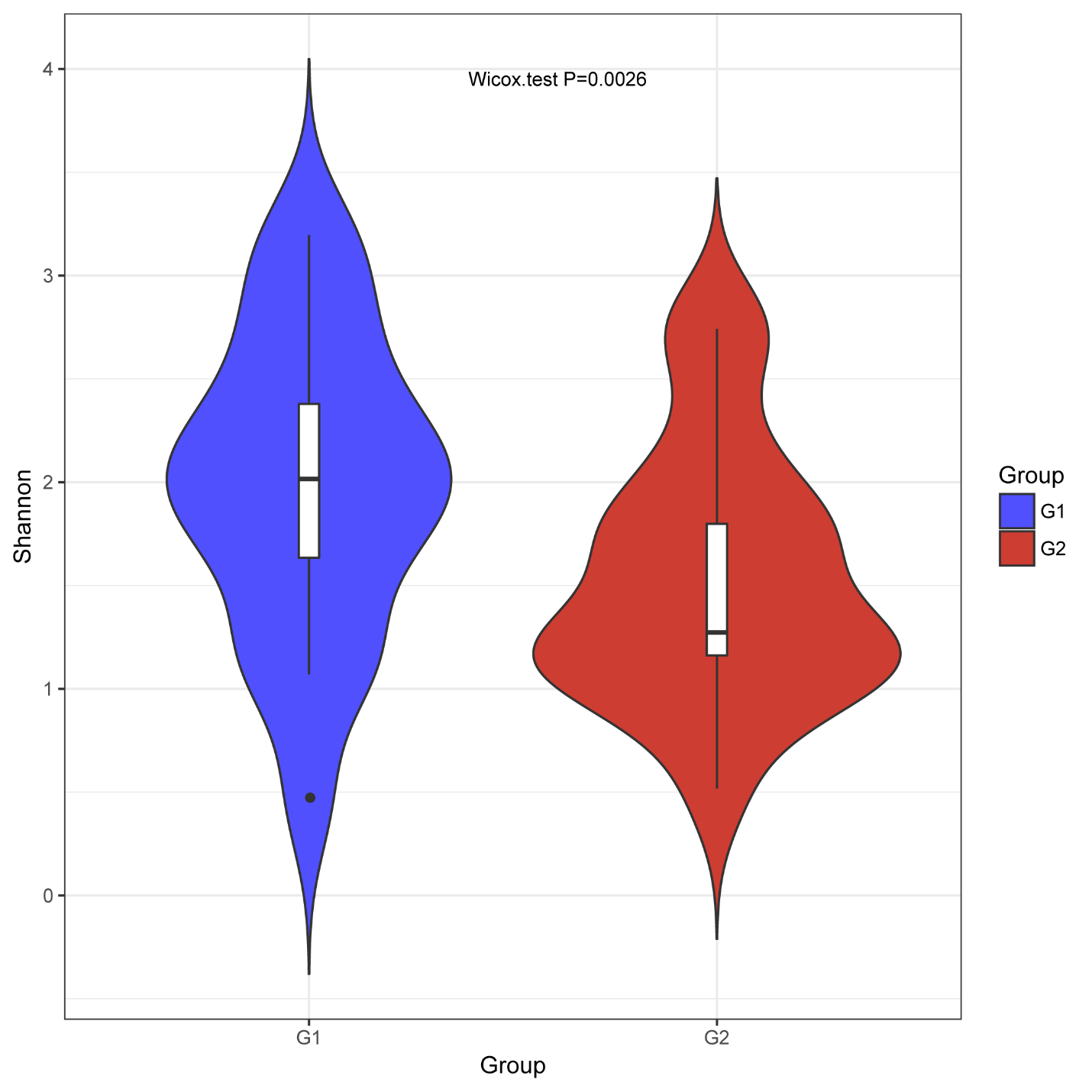


**Figure S2.** **Significant differences in richness diversity estimates of the microbial communities between the two crews.**


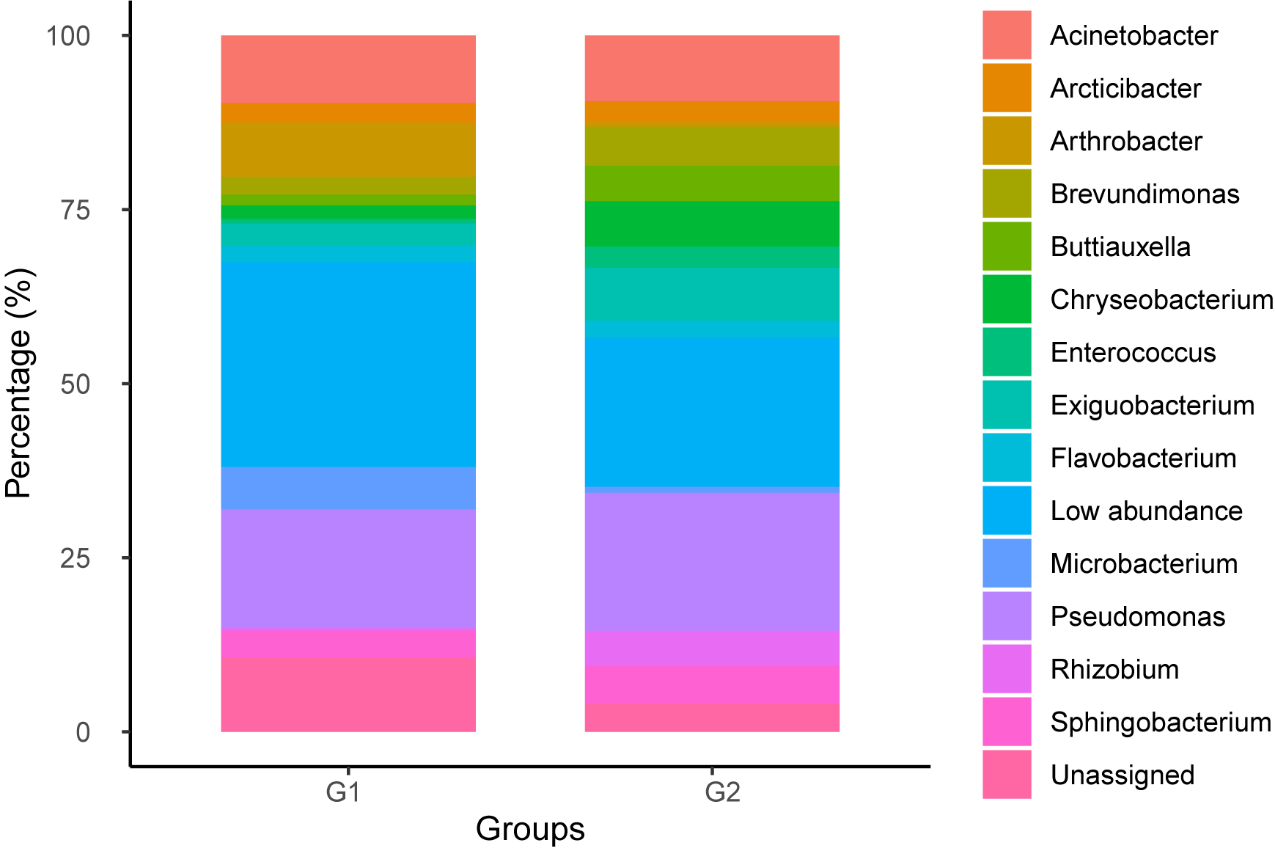


**Figure S3.** **Relative sequence abundance, based on amplicon sequencing, of bacterial genera associated with G1 and G2.**


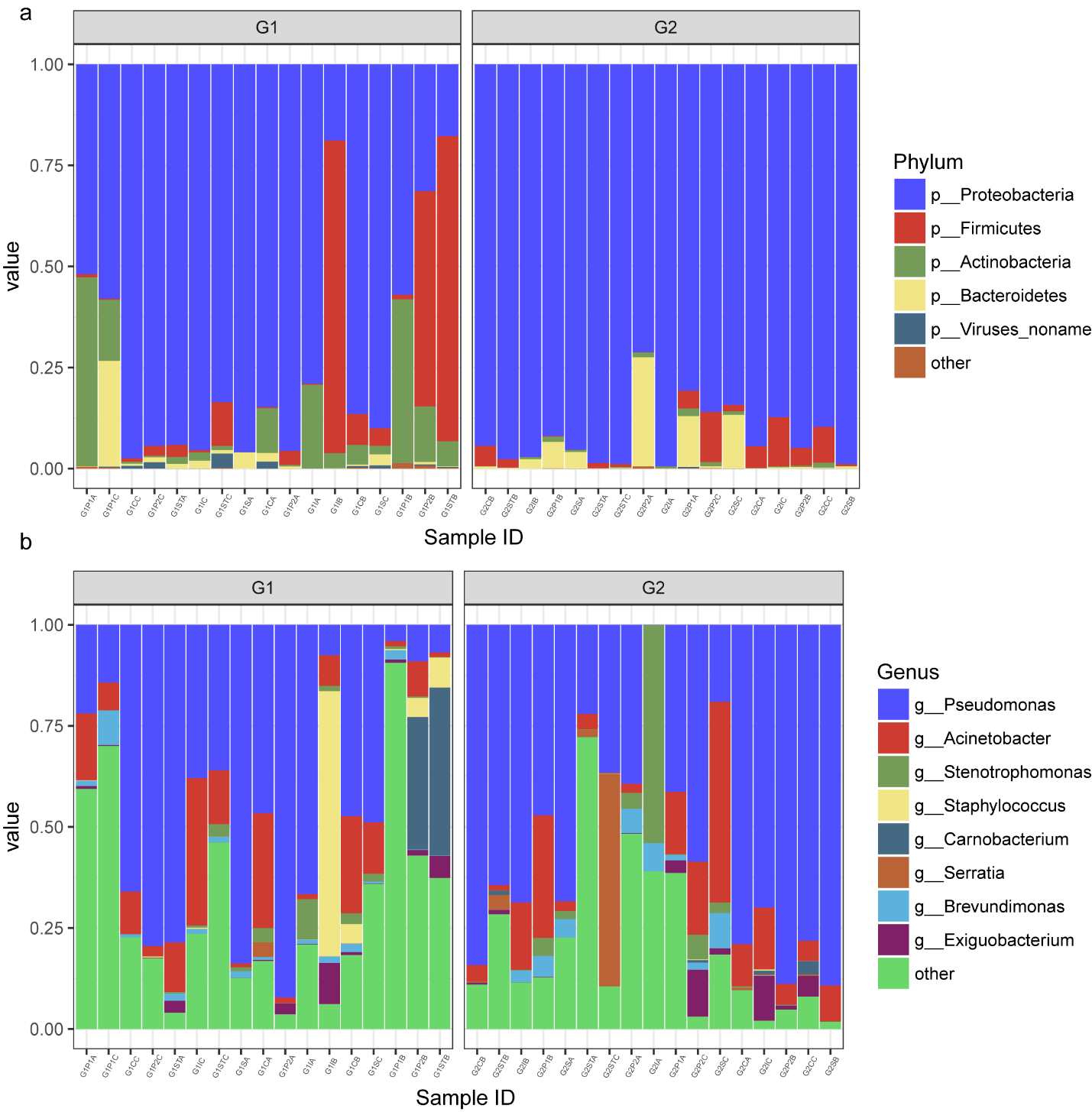


**Figure S4.** **Relative sequence abundance, based on metagenome sequencing, of bacterial phyla (a) and genera (b) associated with G1 and G2.**


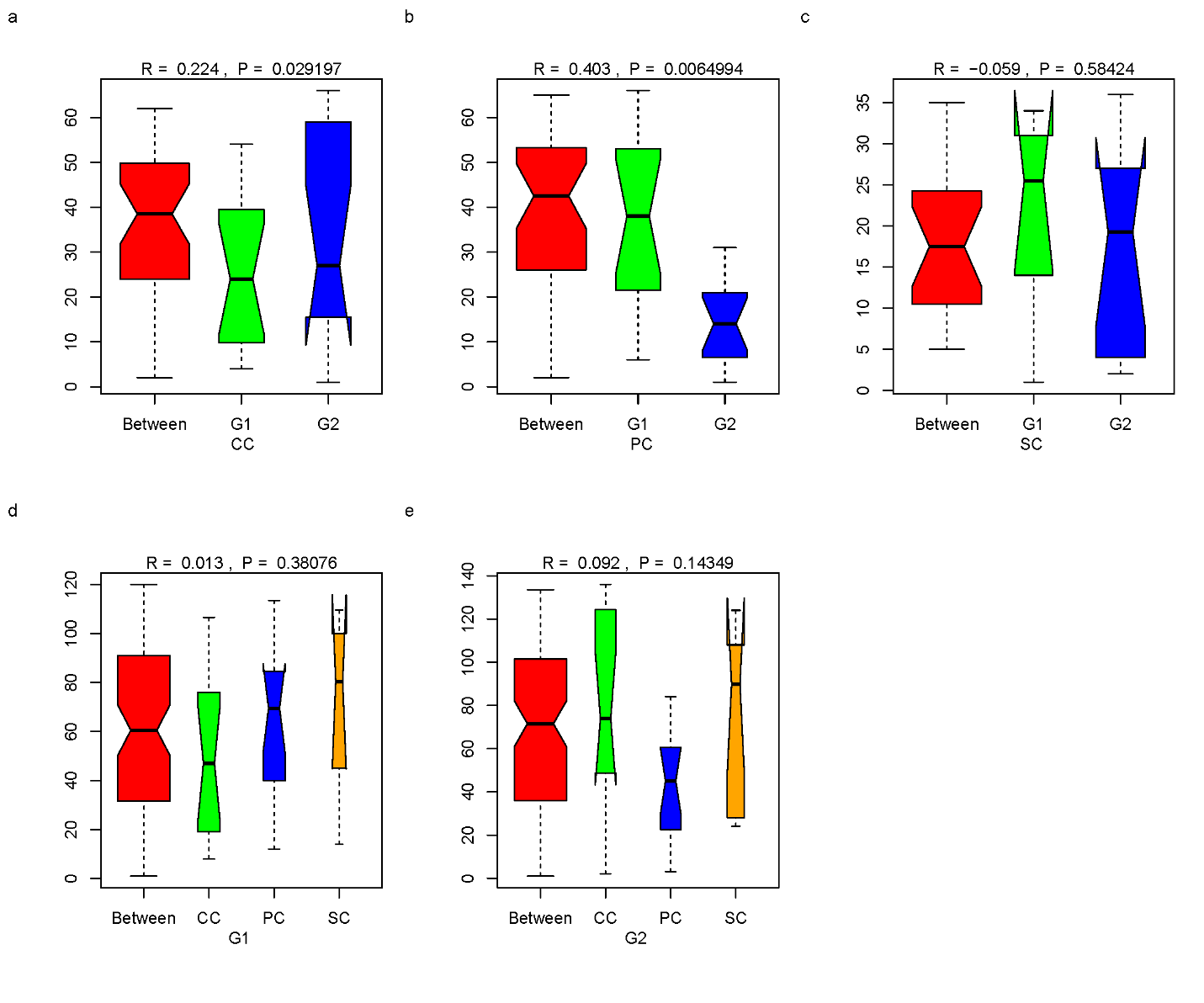


**Figure S5.** **Comparison of community diversity among the different occupant groups and different sampling locations. (a)** Comparison of community diversity among the different occupant groups in the CC. **(b)** Comparison of community diversity among the different occupant groups in the PC. **(c)** Comparison of community diversity among the different occupant groups in the SC. **(d)** Comparison of community diversity among the different sampling sites during the G1 group. **(e)** Comparison of community diversity among the different sampling sites during the G2 group. CC, comprehensive cabin; PC, plant cabin; SC, solid waste treatment cabin.


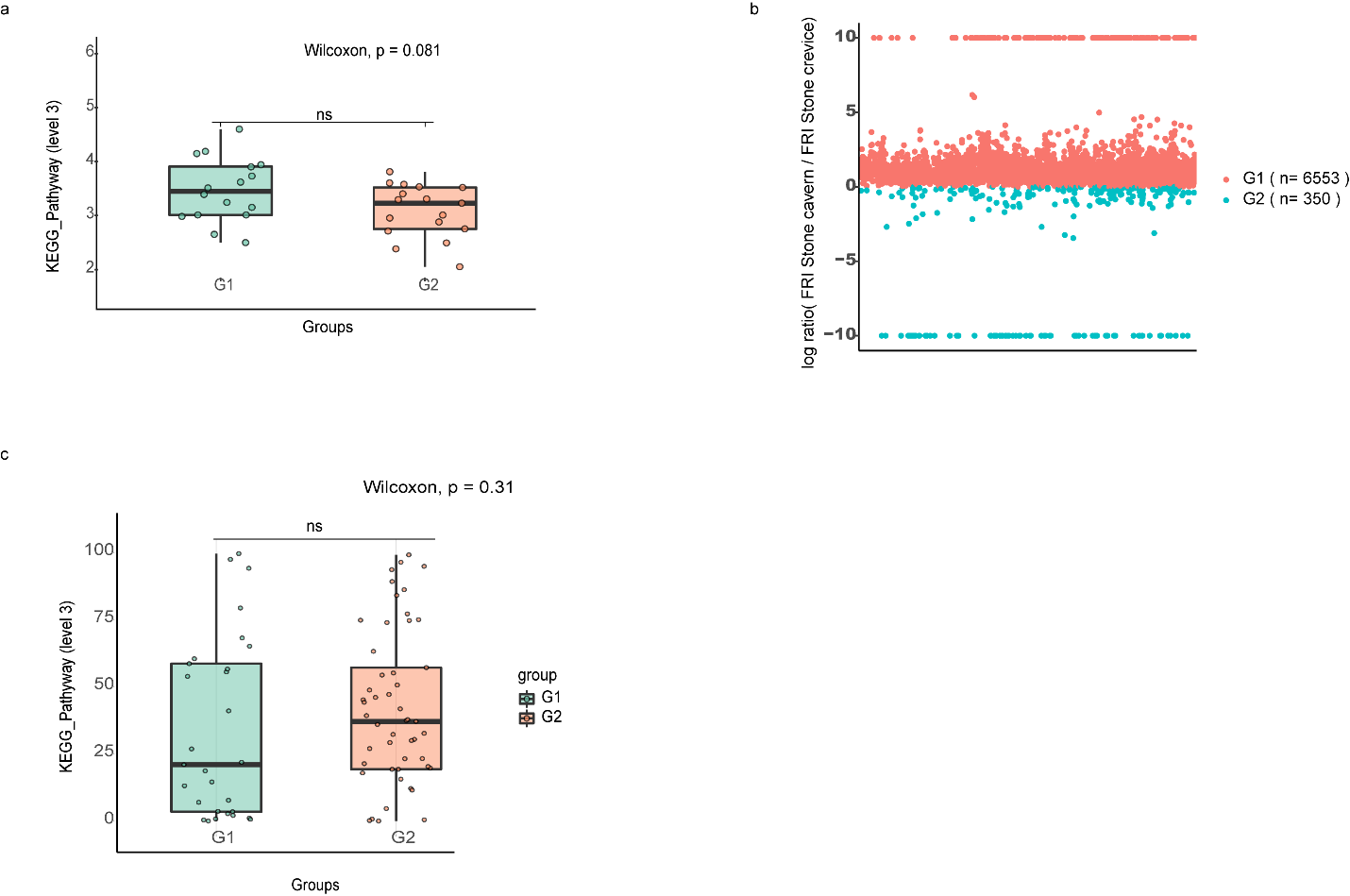


**Figure S6. Microbial functional diversity estimates for the two shifts.** **(a)** Functional diversity between the two crew groups (no significant difference). **(b)** Functional redundancy index. **(c)** Functional diversity analysis based on metagenomics between the two crew groups.


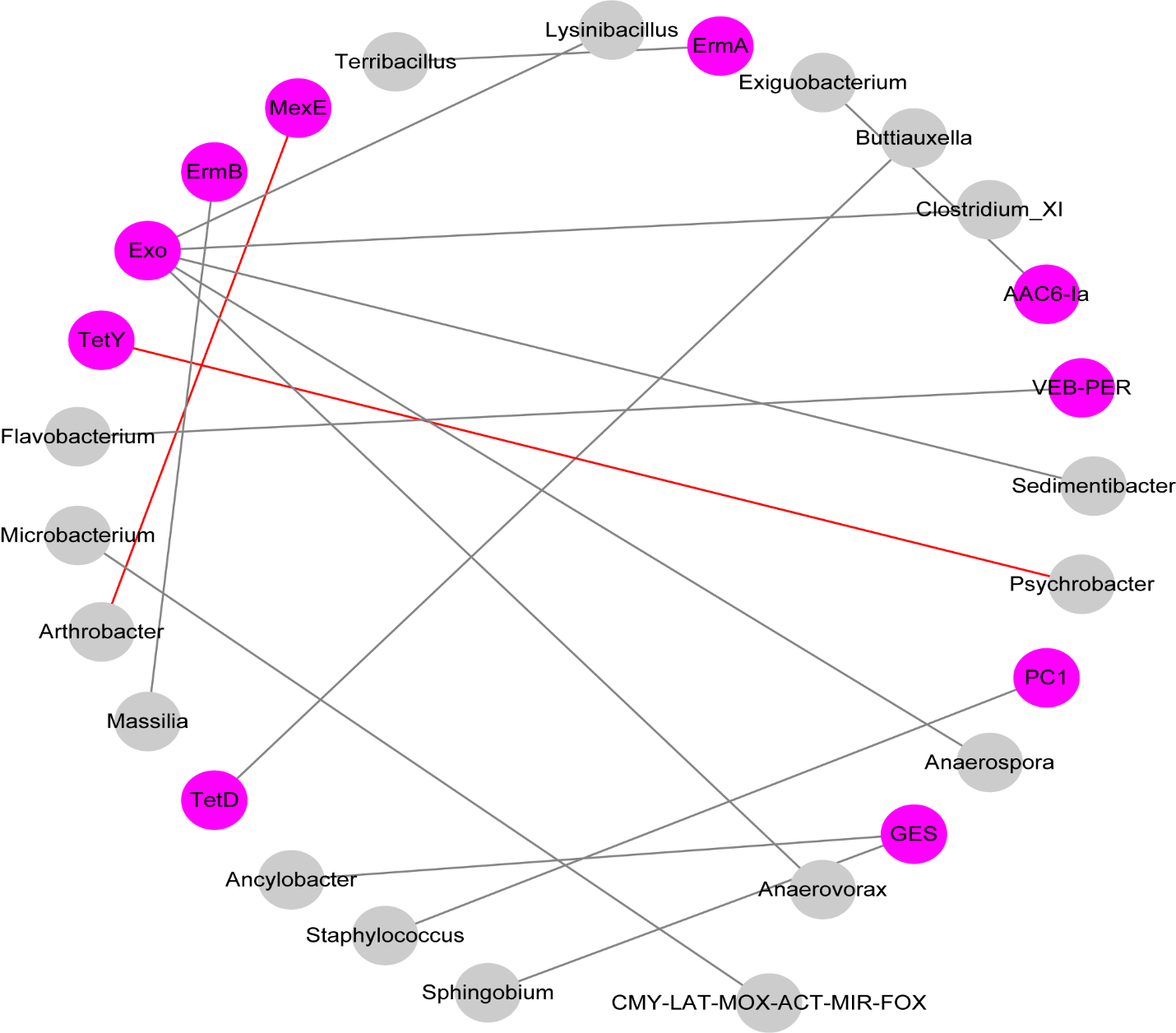


**Figure S7. Correlation network analysis between ARGs and the airborne microbiome (P < 0.05, Spearman’s coefficient > 0.4).** Grey lines between the nodes indicate positive connections between the genera, while red lines indicate negative correlations.
